# Neutrophil count as a reliable marker for diabetic kidney disease in autoimmune diabetes

**DOI:** 10.1186/s12902-020-00597-2

**Published:** 2020-10-22

**Authors:** Yao Yu, Qiuqiu Lin, Dewei Ye, Yanfei Wang, Binbin He, Yanhua Li, Gan Huang, Zhiguang Zhou, Yang Xiao

**Affiliations:** 1 Department of Metabolism & Endocrinology, The Second Xiangya Hospital, Central South University, 139 Renmin Road, Changsha, 410011 Hunan China; 2 Key Laboratory of Diabetes Immunology (Central South University), Ministry of Education; National Clinical Research Center for Metabolic Diseases, Changsha, Hunan China; 3 Joint Laboratory between Guangdong and Hong Kong on Metabolic Diseases, Guangdong Pharmaceutical University, Guangzhou, China; 4 Department of Endocrinology, The First People’s Hospital of Foshan, Foshan, Guangdong China

**Keywords:** Neutrophil, Autoimmune diabetes, Type 1 diabetes, Latent autoimmune diabetes in adults, Diabetic kidney disease

## Abstract

**Background:**

A growing body of evidence supports neutrophils as having an active role in the development of diabetic kidney disease (DKD). However, the clinical relevance of neutrophils and DKD in autoimmune diabetes remains unknown. This study aimed to investigate the relationship between circulating neutrophils and DKD in autoimmune diabetes.

**Methods:**

Patients with type 1 diabetes (T1D, *n* = 226) and latent autoimmune diabetes in adults (LADA, *n* = 79) were enrolled and stratified according to the urinary albumin to creatinine ratio (ACR). Circulating levels of white blood cells (WBCs), including neutrophils, were measured in a central laboratory, and the neutrophil-to-lymphocyte ratio (NLR) was calculated. The risk factors associated with DKD were analysed by logistic regression.

**Results:**

In T1D and LADA patients, the peripheral neutrophil counts increased in parallel with DKD advancement. The neutrophil counts in the patients with macroalbuminuria were significantly higher than those in the patients with normoalbuminuria for each type of diabetes. Furthermore, neutrophil counts positively correlated with ACR in T1D. In addition, neutrophils were independently associated with DKD in T1D in the logistic regression analysis, when various well-known risk factors, including age, gender, disease duration, hypertension, dyslipidemia and smoking status, were adjusted.

**Conclusions:**

Neutrophil counts are closely associated with DKD in patients with autoimmune diabetes, suggesting that neutrophil-mediated inflammation may be involved in the pathogenesis of DKD in patients with autoimmune diabetes.

## Background

Diabetic kidney disease (DKD) is one of the most common chronic complications of diabetes. In recent years, as the prevalence of diabetes has increased year by year, the prevalence of DKD has also increased significantly. More than one-third of diabetes patients suffer from DKD. DKD is the main cause of end-stage renal disease (ESRD), and it is also a high-risk factor for cardiovascular events such as coronary heart disease and stroke. In recent years, in addition to glucose and lipid metabolism disorders and haemodynamic abnormality, studies have shown that multiple pathological processes, such as chronic inflammation, oxidative stress and fibrosis, are also involved in the development of DKD.

The hyperglycaemia status of diabetic patients has long been considered to be the initiating factor of DKD. Glucose and lipid metabolism disorders and renal haemodynamic abnormality caused by hyperglycaemia are the two major pathological bases of DKD. Based on these factors, the current treatment of DKD mainly aims at controlling blood glucose, blood lipids, and blood pressure and improving renal haemodynamics.

However, comprehensive management that aims to maintain blood glucose, blood lipids and blood pressure within normal levels cannot completely prevent the development of DKD. Therefore, in addition to the above two aspects, there are other pathological processes involved in the development of DKD. In recent years, studies have demonstrated that a large number of lymphocytes, macrophages and mast cells accumulate in the kidney tissue of patients with DKD, which secrete large amounts of inflammatory mediators, cytokines and oxygen free radicals, directly or indirectly leading to kidney damage and increased renal fibrosis. The inflammatory hypothesis suggests that metabolic disorders in DKD patients activate inflammatory signals in the body, which in turn causes deposition of extracellular matrix in the kidney and promotes fibrosis. Therefore, inflammation plays an important role in the pathogenesis of DKD [1]. In the circulation, neutrophils are the main immune cell type associated with the inflammatory response, and increasing numbers of studies suggest that the inflammation they mediate plays a role in DKD.

Neutrophils may migrate to the kidney by increasing spontaneous adhesion [2], followed by abnormal activation of the secretion of proinflammatory cytokines, degranulated products, and reactive oxygen species (ROS) [3], further damaging the kidneys. Clinical studies have found that peripheral total white blood cells (WBCs), neutrophils, and the Neutrophil-to-lymphocyte ratio (NLR) are independently associated with DKD in T2D [3–11]. However, the association between these factors and autoimmune diabetes, such as T1D and LADA, patients has not yet been explored. Thus, the purpose of this study was to investigate the relationship between neutrophils and DKD in patients with autoimmune diabetes and to explore new directions for the prevention and treatment of DKD.

## Methods

### Subjects

We retrospectively studied the clinical data from 305 patients with autoimmune diabetes, including 226 with T1D and 79 with LADA, who visited the Department of Endocrinology and Metabolism, the Second Xiangya Hospital of Central South University, between December 2009 and May 2018. We recorded confidential information on anthropometric measurements, biochemical parameters and medication history. Patients with any acute or chronic infections, any history of malignant tumours or with other kidney, severe liver, cardiovascular and cerebrovascular diseases were excluded from this study. T1D was diagnosed according to the standards of the American Diabetes Association. Patients with LADA were recruited in the study according to the inclusion criteria previously described [12]. Definition of hypertension and dyslipidemia was reported previously [13]. Smoking was defined as those who have smoked now or before, while non-smokers are those who have never smoked.

Age, gender, waist circles (WC), body mass index (BMI), blood pressure, disease duration, fasting glucose (FBG), 2 h postprandial glucose (PBG), fasting C-peptide (FCP), 2 h postprandial C-peptide (PCP), haemoglobin A1C (HbA1c), lipid profiles, liver function, and renal function were collected from the electronic medical records of our department, and the measurement methods were previously published [13]. Estimated glomerular filtration rate (eGFR) was calculated using the standard Chronic Kidney Disease Epidemiology Collaboration formula (CKD-EPI) for each patient. Patients with an eGFR less than 30 mL/min/1.73 m^2^ were excluded.

Peripheral blood leukocyte analysis included total WBC counts and absolute counts of neutrophils, monocytes, lymphocytes, eosinophils, and basophils. To reduce the confounding effects of infection, the WBC counts ranging from 4.0*10^9^/l to 10.0*10^9^/l and neutrophil counts in the normal range were taken into account for the analysis of the included patients. All abnormal or atypical specimens of WBCs and neutrophils were excluded.

Fresh morning spot urine samples were obtained from all patients, and the Urinary albumin to creatinine ratio (ACR) (mg/g) was calculated by dividing microalbumin (mg/dl) by creatinine (g/dl). According to their ACRs, the diabetic patients were divided into three groups: normoalbuminuria (ACR < 30 mg/g), microalbuminuria (ACR 30–300 mg/g), and macroalbuminuria (ACR > 300 mg/g). DKD was accepted as either micro/macroalbuminuria, eGFR < 75 ml/min/1.73 m^2^ or both [14]. The study excluded patients with known causes of kidney disease other than diabetes.

The study was approved by the Ethics Committee of the Second Xiangya Hospital, Central South University. The study was conducted in accordance with the principles of the Helsinki Declaration. Due to the retrospective nature of the study, the requirement to obtain informed consent was waived.

### Statistical analysis

All analyses were performed using IBM Statistical Product and Service Solutions (SPSS) statistics 23.0 software (Armonk, N.Y., USA). The Kolmogorov-Smirnov test was used to test the data for normality. The normally distributed data were expressed as the means ± standard deviations and were compared with one-way ANOVA between groups. The non-normally distributed data are expressed as the medians and interquartile ranges and were naturally logarithmically transformed before analysis. For continuous data with a non-normal distribution, the values were compared between groups with the Kruskal-Wallis test. The Pearson correlation coefficient was calculated to estimate the correlations between variables. The risk factors for DKD were identified by binary logistic regression analysis. The results are expressed as odds ratios (ORs) and 95% confidence intervals (CIs). In all statistical comparisons, *P* < 0.05 was considered statistically significant.

## Results

The study included 226 patients with T1D and 79 patients with LADA. Age, BMI, systolic blood pressure (SBP), diastolic blood pressure (DBP), HbA1c, FCP, PCP, triglyceride (TG) and ACR of LADA patients were higher compared with T1D patients (for TG, *P* = 0.005; for others, *P* < 0.001). However, eGFR and high density lipoprotein cholesterol (HDL-c) levels of LADA patients were lower than those of T1D patients (for eGFR, P < 0.001; for HDL-c, *P* = 0.001).

Among 226 patients with T1D, 204 had normoalbuminuria, 10 had microalbuminuria, and 12 had macroalbuminuria. Among 79 patients with LADA, 61 had normoalbuminuria, 10 had microalbuminuria, and 8 had macroalbuminuria. The characteristics of these patients are shown in Table 1. In the patients with T1D or LADA, duration of diabetes, SBP and uric acid were higher in the patients with macroalbuminuria than in those with normoalbuminuria (*P* < 0.05 for all). In the two groups of diabetic patients, the levels of eGFR and serum albumin gradually decreased parallel to the severity of albuminuria (P < 0.01). In patients with T1D, there was a significant trend in age and DBP across the three albuminuria groups (*P* < 0.05). However, no similar changes were observed in the patients with LADA (Table 1).

**Table 1:** The characteristics of diabetic patients

	T1D			*P*	LADA			*P*
	Normoalbuminuria	Microalbuminuria	Macroalbuminuria		Normoalbuminuria	Microalbuminuria	Macroalbuminuria	
Number	204	10	12		61	10	8	
Age (yr)	21.67±14.92	28.50±28.66	33.50±17.06*	**0.017**	52.46±10.48	58.90±14.14	57.00±9.26	0.156
Gender (male/female)	100/104	3/7	5/7	0.456	35/26	7/3	4/4	0.667
Duration of diabetes (yr)	5.13±5.05	7.27±7.59	11.03±5.12*	**<0.001**	5.42±5.45	10.40±5.99*	12.56±6.54*	**0.001**
BMI (kg/m²)	18.95±3.20	19.00±2.73	21.01±2.31*	0.110	21.59±2.79	22.86±3.62	21.96±2.42	0.429
Smoker (%)	35.3	40.0	41.7	0.870	24.6	50.0	37.5	0.231
WHR	0.84±0.07	0.83±0.08	0.87±0.07	0.296	0.89±0.06	0.91±0.05	0.88±0.06	0.530
SBP (mmHg)	112.94±15.53	114.20±20.91	139.42±34.21*∆	**<0.001**	122.72±17.23	133.90±22.83	137.75±24.26*	**0.039**
DBP (mmHg)	70.56±12.69	75.00±16.21	81.67±16.46*	**0.012**	77.00±9.82	79.90±15.50	85.13±10.74	0.121
HbA1c (%)	8.37±2.19	7.63±1.85	7.48±1.40	0.228	9.45±2.35	9.69±2.53	9.89±2.17	0.864
FBG (mmol/l)	9.17±4.56	7.60±3.08	10.63±4.78	0.315	8.31±4.31	8.07±3.55	6.56±3.73	0.579
PBG (mmol/l)	16.01±7.17	14.88±6.84	13.27±6.56	0.409	13.50±6.18	12.66±4.92	11.81±5.81	0.746
FCP (pmol/l)a	25.05 (5.50, 89.58)	10.05 (5.50,44.18)	5.50 (5.50,157.50)	0.405	90.00 (30.85,163.75)	136.75 (37.53,224.88)	70.55 (9.48, 188.48)	0.771
PCP (pmol/l) a	41.00 (5.70,215.08)	30.60 (5.85,86.50)	17.1 (5.50,126.30)	0.512	179.90 (56.50,312.78)	251.65 (76.80,593.80)	202.30 (13.80,255.23)	0.778
TG (mmol/l) a	0.74 (0.58,1.11)	0.86 (0.49,1.23)	0.84 (0.65,1.25)	0.422	0.93 (0.68, 1.17)	1.31 (0.86, 1.70)	1.30 (0.91, 1.54)	0.068
TC (mmol/l)	4.28±0.91	4.15±0.83	4.63±1.26	0.416	4.19±0.72	4.07±0.82	3.94±0.98	0.680
LDL-c (mmol/l)	2.42±0.78	2.24±0.83	2.72±1.04	0.354	2.43±0.64	2.38±0.78	2.37±0.94	0.965
HDL-c (mmol/l) a	1.45 (1.20,1.67)	1.55 (1.35,1.75)	1.48 (1.30,1.64)	0.574	1.29 (1.13, 1.59)	1.34 (0.91, 1.42)	1.18 (0.94, 1.45)	0.241
Albumin (g/l))	42.37±4.08	41.72±3.65	36.02±7.87*∆	**<0.001**	37.44±2.96	36.38±2.91	33.21±3.92*∆	**0.003**
TBIL (umol/l)	12.26±5.35	11.68±3.62	9.46±4.00	0.194	12.14±4.40	10.78±5.84	7.92±2.74	0.081
DBIL (umol/l)	3.92±1.94	3.87±1.08	3.13±1.66	0.382	3.88±1.44	3.28±1.65	2.64±1.18	0.073
UA (umol/l)	255.92±69.79	289.92±78.39	312.02±95.63*	**0.018**	230.89±53.26	303.57±63.54*	351.35±92.66*	**<0.001**
EGFR (ml/(min*1.73m²))a	139.15 (111.45-168.47)	110.36 (72.18-135.87)*	57.24 (50.42-116.46)*	**0.001**	102.23 (80.56-124.21)	72.70 (47.20-144.44)	59.29 (42.45-96.85)*	**0.022**
No medication	0	0	0		0	0	0	
Oral hypoglycemic agent	0	0	0		4	1	0	
Insulin	204	10	12		29	4	4	
Oral hypoglycemic agent + insulin	0	0	0		28	5	4	

Data are expressed as mean ± SD or median (interquartile range) as appropriate. Compared with the normalalbuminuria group: *p < 0.05; Compared with the microalbuminuria group: ∆ *p* < 0.05; aNon-parametric kruskal-wallis method was used to test the differences among the three groups.

*Abbreviations*: *T1D* type 1 diabetes, *LADA* latent autoimmune diabetes in adults, *BMI* body mass index, *WHR* waist to hip ratio, *TG* triglycerides, *TC* total cholesterol, *HDL-c* high density lipoprotein cholesterol, *LDL-c* low density lipoprotein cholesterol, *TBIL* total bilirubin, *DBIL* direct bilirubin, *EGFR* estimated glomerular filtration rate, *SBP* systolic blood pressure, *DBP* diastolic blood pressure, *FBG* fasting blood glucose, *PBG* 2h postprandial blood glucose, *FCP* fasting C-peptide, *PCP* 2h postprandial C-peptide, *UA* uric acid

**Fig. 1:**
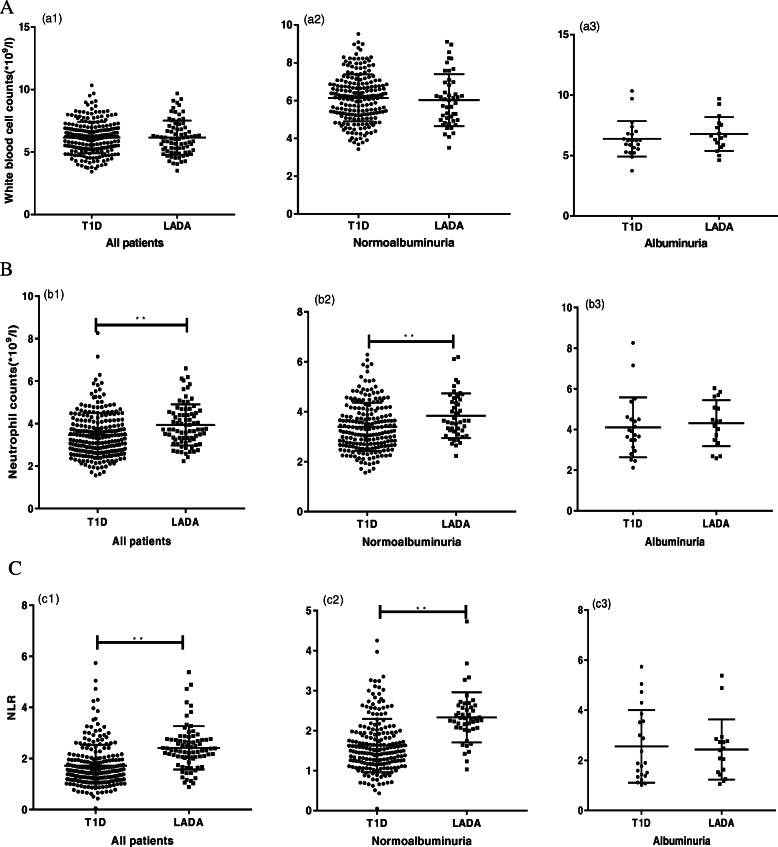
WBCs, neutrophil counts and NLR in autoimmune patients. WBCs (**a**), neutrophil counts (**b**) and NLR (**c**) were compared between T1D and LADA patients for all, with normoalbuminuria or albuminuria. The horizontal line indicates the median and quartiles. * *P* < 0.05; ** *P* < 0.01

The total WBC, neutrophil counts and the NLRs of the different types of diabetic patients were compared in different status of albuminuria (Fig. 1a, b, c). For the total WBC counts of all patients, there were no significant differences between T1D and LADA. For the neutrophil counts, in all patients and in the normoalbuminuria patients, the patients with LADA had higher neutrophil counts than the patients with T1D (*P* < 0.05). For the NLR, in all patients and in the normoalbuminuria patients, the NLR of the patients with LADA was significantly higher than that of the patients with T1D (*P* < 0.01) (Fig. 1).

**Fig. 2:**
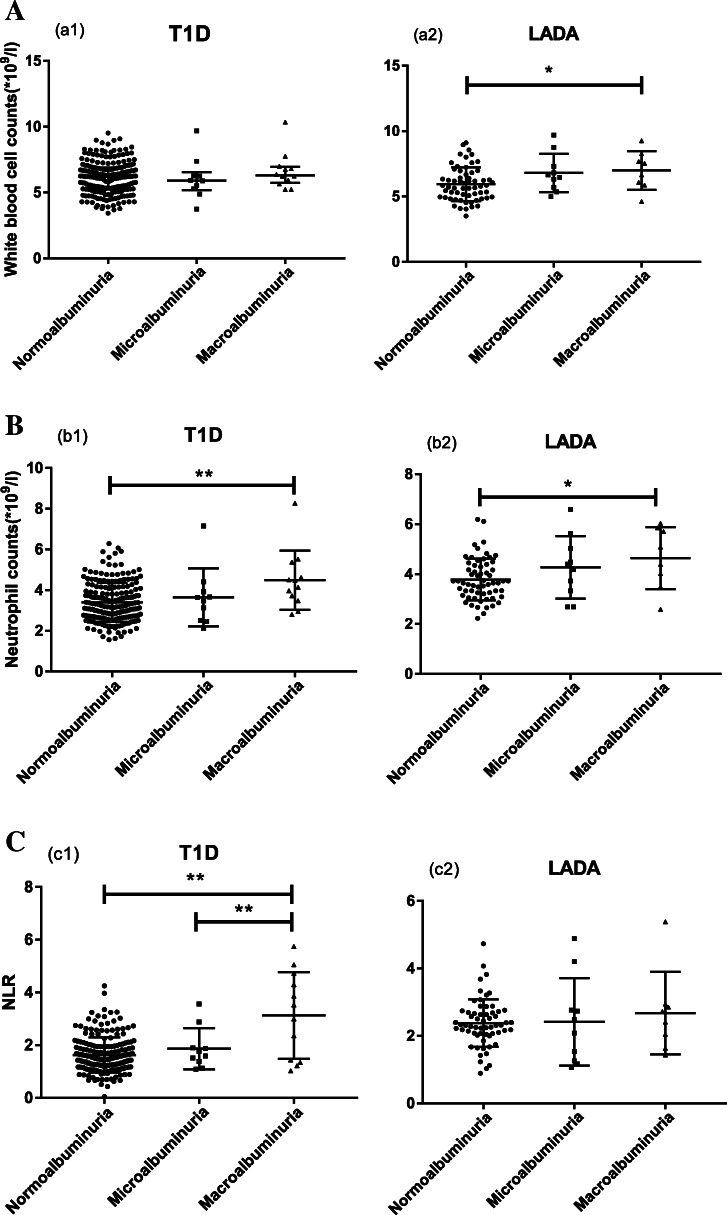
WBCs, neutrophil counts and NLR in autoimmune diabetes with different albuminuria status. WBCs (**a**), neutrophil counts (**b**) and NLR (**c**) were compared among normoalbuminuria, microalbuminuria and macroalbuminuria group in T1D and LADA patients. The horizontal line indicates the median and quartiles. * *P* < 0.05; ** *P* < 0.01

Within each diabetic type group, the WBC counts, neutrophil counts and NLR were analysed between the different albuminuria groups (Fig. 2). The WBC counts in the patients with LADA gradually increased from normoalbuminuria, microalbuminuria to macroalbuminuria. Among the patients with LADA, WBC counts in the microalbuminuria and macroalbuminuria groups were significantly greater than those in the normoalbuminuria group (Fig. 2, *P* < 0.05). The neutrophil counts in the macroalbuminuria groups with T1D or LADA were significantly greater than those in the corresponding normoalbuminuria group (Fig. 2, *P* < 0.01, P < 0.05, respectively). The NLR of the macroalbuminuria group among the patients with T1D was higher than those of the normoalbuminuria (P < 0.01) and microalbuminuria groups (P < 0.05).

**Fig. 3:**
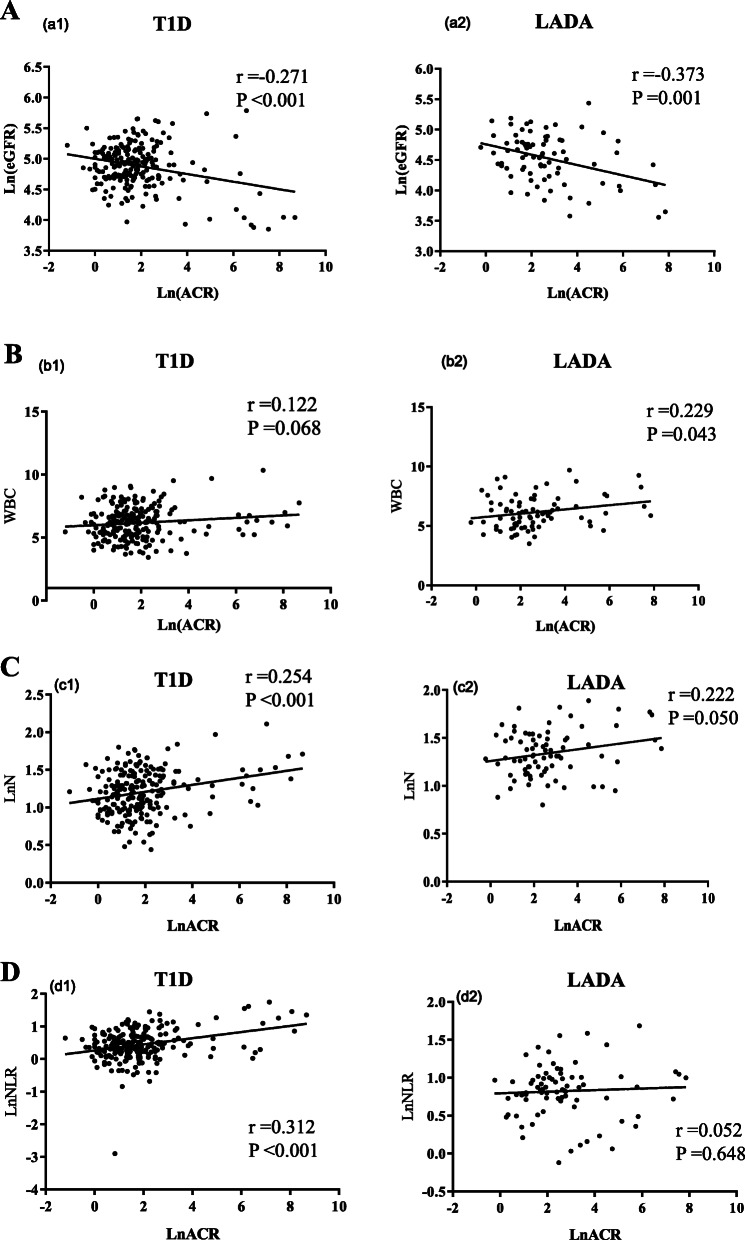
Correlations between ACR and other parameters. Correlations between ACR and eGFR (**a**), WBCs (**b**), neutrophil (**c**) or NLR (**d**) in T1D and LADA patients. Ln, Natural logarithm

In T1D, ACR was negatively correlated with eGFR, while ACR was positively correlated with neutrophil counts and NLR (Fig. 3, *P* < 0.001). WBC, neutrophil counts and NLR were all negatively correlated with the HDL-c level. The neutrophil counts and the NLR were positively correlated with disease duration, age, BMI, SBP, DBP and creatinine, but negatively correlated with albumin levels.. WBC and neutrophil counts were positively associated with TG, low density lipoprotein cholesterol (LDL-c), and negatively associated with PCP. There was positive correlation between NLR and the eGFR (Table 2).

**Table 2:** Correlation between WBC, neutrophil count, NLR and metabolic variables

		T1D			LADA		
		WBC	N^a^	NLR^a^	WBC	N^a^	NLR^a^
Duration	r	0.097	0.225**	0.287**	0.153	0.244*	0.205
Age	r	0.023	0.270**	0.464**	-0.060	0.066	0.296**
WHR	r	0.091	0.080	0.089	0.021	0.051	0.103
BMI	r	0.097	0.286**	0.341**	0.227*	0.201	-0.005
SBP	r	0.007	0.249**	0.371**	0.241*	0.188	-0.067
DBP	r	-0.002	0.196**	0.287**	0.227*	0.081	-0.228*
FBG	r	0.058	0.102	0.078	0.057	0.056	0.031
PBG	r	-0.011	-0.078	-0.124	0.010	0.017	0.035
FCP^a^	r	-0.135	-0.110	-0.089	0.037	0.056	0.049
PCP^a^	r	-0.154*	-0.143*	-0.119	0.005	0.009	0.020
HbA1C	r	0.104	0.065	-0.002	-0.100	-0.087	-0.011
TC	r	0.123	0.120	0.046	0.069	0.078	0.078
TG^a^	r	0.138*	0.155*	0.069	0.176	0.198	0.099
LDL-c	r	0.190**	0.206**	0.110	0.207	0.193	0.060
HDL-c^a^	r	-0.143*	-0.190**	-0.153*	-0.218	-0.202	<0.001
Gender	r	0.012	-0.015	0.134*	-0.126	-0.138	-0.161
Smoking	r	-0.001	-0.019	0.030	0.245*	0.173	-0.038
Cr	r	0.001	0.216**	0.345**	0.112	0.205	0.221
Albumin	r	-0.055	-0.207**	-0.364**	-0.075	-0.188	-0.185
TBIL	r	0.003	-0.023	-0.104	-0.216	-0.248*	-0.021
DBIL	r	-0.017	-0.035	-0.093	-0.185	-0.219	-0.023
UA	r	0.020	0.055	0.091	0.337**	0.370**	0.161
eGFR^a^	r	0.023	-0.074	-0.168**	0.047	-0.076	-0.231*

*Abbreviations*: *WBC* white blood cell, *N* neutrophil, *NLR* neutrophil to lymphocyte ratio, *BMI* body mass index, *WHR* waist to hip ratio, *SBP* Systolic blood pressure, *DBP* Diastolic blood pressure, *FBG* fasting blood glucose, *PBG* 2h postprandial blood glucose, *FCP* Fasting C-peptide, *PCP* postprandial C-peptide, *TC* Total cholesterol, *TG* triglycerides, *HDL-c* high density lipoprotein cholesterol, *LDL-c* low density lipoprotein cholesterol, *Cr* creatinine, *TBIL* total bilirubin, *DBIL* direct bilirubin, *UA* uric acid, *ACR* urinary albumin to creatinine ratio, *EGFR* estimated glomerular filtration rate; *p < 0.05;**p < 0.01. ^a^Log transformed before analysis

In LADA, ACR was negatively correlated with eGFR, while ACR was positively correlated with WBC counts (Fig. 3, *P* = 0.001 and *P* = 0.043, respectively). There were positive correlations between WBC and BMI, SBP, DBP, smoking and uric acid. Neutrophil counts were positively associated with disease duration and uric acid, but negatively associated with total bilirubin. Moreover, NLR showed positive correlation with age and eGFR, and negative correlation with DBP (Table 2).

In T1D, univariate analyses were performed and demonstrated that age, disease duration and neutrophil counts were significantly related to DKD (Table 3). Logistic regression analysis was conducted where the stages of DKD was used as the dependent variable, while age, gender, disease duration, smoking status, BMI, hypertension, dyslipidemia, FBG, HbA1c, FCP and neutrophil counts were used as independent variables. The result revealed that age (*P* = 0.001), female gender (*P* = 0.025), HbA1c (*P* = 0.018), and neutrophil counts (*P* = 0.043) were independent risk factors for DKD in T1D (Table 3). In LADA, univariate analyses proved that age, disease duration and hypertension were associated with DKD (Table 3). Logistic regression showed that, after adjustment for the conventional risk factors mentioned above, age (*P* = 0.007), disease duration (*P* = 0.041) and dyslipidemia (*P* = 0.019) were independent risk factors for DKD in LADA (Table 3).

**Table 3:** Binary logistic regression analysis found risk factors for diabetic kidney disease

	T1D					LADA				
	Simple	Multiple				Simple	Multiple			
	*P*	OR	*P*	95%CI		*P*	OR	*P*	95%CI	
				LCI	UCI				LCI	UCI
Age	<0.001**	1.061	0.001**	1.023	1.101	<0.001^**^	1.140	0.007**	1.037	1.253
Gender	0.237	4.278	0.025*	1.195	15.309	0.373	4.195	0.131	0.651	27.012
Duration	<0.001**	1.060	0.201	0.969	1.159	0.001^**^	1.177	0.041*	1.006	1.376
BMI	0.303	0.916	0.411	0.742	1.130	0.581	0.763	0.078	0.565	1.030
HbA1c	0.084	0.586	0.018*	0.376	0.913	0.811	0.838	0.405	0.554	1.270
FBG	0.741	1.010	0.892	0.880	1.159	0.390	0.936	0.516	0.767	1.143
FCP	0.694	1.000	0.898	0.993	1.008	0.528	1.000	0.943	0.994	1.006
Hypertension	0.445	0.659	0.464	0.216	2.008	0.011*	4.678	0.109	0.711	30.792
Dyslipidemia	0.501	0.801	0.719	0.240	2.673	0.404	8.378	0.019*	1.410	49.779
N	0.003**	1.659	0.043*	1.017	2.706	0.155	0.622	0.331	0.238	1.621
Smoking	0.772	1.988	0.231	0.646	6.119	0.820	4.671	0.135	0.620	35.209

*Abbreviations*: *T1D* type 1 diabetes, *LADA* latent autoimmune diabetes in adults, *N* neutrophil counts, *BMI* body mass index, *FBG* fasting blood glucose, *FCP* fasting C-peptide; *p < 0.05; **p < 0.01

The patients with DKD were divided in two groups: albuminuric DKD (A-DKD) – patients with albuminuria and non-albuminuric DKD (NA-DKD) - patients with eGFR < 75 mL/min and without albuminuria. For A-DKD, the multivariable analysis (logistic regression), adjusted to age, gender, disease duration, smoking status, BMI, hypertension, dyslipidemia, FBG, HbA1c, FCP and neutrophil counts, showed that developing A-DKD was associated with HbA1c (*P* = 0.040), and neutrophil counts (*P* = 0.021) in T1D, while it was associated with disease duration (*P* = 0.022) and hypertension (*P* = 0.011) in LADA (Supplementary Table 1). For NA-DKD, on a similar logistic regression model, there was a trend of age to be significantly associated with NA-DKD in T1D (*P* = 0.057), while age (*P* = 0.016), female gender (*P* = 0.036) and hypertension (*P* = 0.048) were independently associated with NA-DKD in LADA (Supplementary Table 2).

## Discussion

Our study provided the first evidence that in patients with T1D or LADA, the peripheral total WBC counts, neutrophil counts, and NLR, even within the normal range, were associated with DKD. The association between neutrophils and DKD remained even after controlling for conventional risk factors, including age, gender, smoking status, hypertension, dyslipidemia, obesity and glucose control.

DKD is common among all types of diabetes; thus, hyperglycaemia is a major risk factor for DKD. However, hyperglycaemia does not account for all changes that are observed in the renal tissue [15]. The pathogenesis of DKD is complex, including genetics, haemodynamic changes, disorders of glucose and lipid metabolism, effects of cytokines and growth factors, oxidative stress, and inflammatory responses. Several lines of evidence demonstrate inflammation as a cardinal pathogenic mechanism corresponding to the development and progression of DKD, in which several types of innate immune cells are actively involved [16]. Neutrophils, as the most abundant and inflammation-related immune cell type in the circulation, may be involved in the pathogenesis of DKD.

The abnormal activation of blood neutrophils has been reported in diabetic patients [17–19]. The oxidation of serum albumin may cause neutrophil activation and further oxidation of albumin in diabetes, which are important in the severity and progression of DKD [19]. Neutrophils from DKD patients exhibited faster exocytosis of primary granules than those from either normal subjects or patients without DKD, and also cannot remove adhesion molecule CD11b from the cell membrane, leading to the persistent increase of CD11b [17]. The high expression of CD11b may play a guiding role in the migration of neutrophils in inflamed kidney tissue, which exerted the up-regulated expression of cell adhesion molecule ICAM-1. Additionally, the metabolic disturbances accompanying the impairment of diabetes control could induce neutrophil adherence to foreign surfaces and superoxide anion production in diabetic patients [2]. Consistent with this, the spontaneous adhesion of neutrophils in diabetic patients with albuminuria was significantly higher than that in diabetic patients with normal albuminuria or healthy controls [18].

The exact molecular mechanism by which neutrophils are involved in the development of DKD is unclear. However, there is some evidence that neutrophils could play a role in this pathological process. The migration of neutrophils to the kidney is a critical step in the progression of DKD. The influx of neutrophils usually results from an acute response to injury or inflammation. Neutrophil secreting enzymes and oxidation products can damage the local microenvironment and lead to tissue injury [20]. Recent studies revealed that neutrophils from T1D patients were primed to produce neutrophil extracellular traps (NETs), which are composed of DNA, histones, and neutrophil proteins, and a high glucose level can induce NETs in vitro [21, 22]. In addition, myeloperoxidase (MPO), a well-established marker of NET formation, was observed to increase in the kidneys of Streptozotocin-induced diabetic rats [23], suggesting that neutrophil-formed NETs may engage in the pathogenic mechanisms of DKD.

Furthermore, studies suggested that NLR may be important as a potential factor for evaluating diabetic patients with a higher degree of albuminuria [24]. Several epidemiological and clinical studies have evaluated the association of neutrophils or the NLR with DKD in T2D. Azab showed that the NLR could act as a predictor of worsening renal function in 338 American diabetic patients [4]. Chung et al. demonstrated that peripheral neutrophil counts were independently and significantly associated with DKD in 1480 Chinese patients with T2D [3]. In another study that recruited 253 Chinese subjects with T2D, an increased NLR was significantly associated with DKD, and the patients had a 2.088-fold increased risk of DKD for every unit increase in the NLR [5]. Afsar et al. reported that in 80 newly diagnosed T2D patients, NLR was independently associated with 24-h urinary protein and urinary albumin excretion [6]. Ciray et al. demonstrated that among the 114 T2D patients, NLR was positively related to microalbuminuria and negatively related to eGFR [7]. Akbas et al. showed that in 200 T2D patients, the level of albuminuria increased with the increase of NLR, and it was found that NLR was independently associated with albuminuria [8]. Kahraman et al. also showed a significant correlation between proteinuria or glomerular filtration rate and NLR in T2D patients [9]. In addition, a longitudinal study showed that NLR could predict renal function loss in 108 PIMA Indians and 941 Europeans with T2D [10]. A recent study recruited 247 T2D patients with DKD confirmed by biopsy, which proved that NLR was significantly associated with interstitial fibrosis, renal tubular atrophy, and renal dysfunction [11].

To the best of our knowledge, our study provided the first clinical evidence addressing the relationship between neutrophil counts and DKD in T1D and LADA patients. We showed that the patients with albuminuria had higher neutrophil counts than the patients without albuminuria in the contexts of T1D and LADA. For all patients or those with normoalbuminuria, the neutrophil counts showed a significant decrease in patients with T1D compared with those with LADA, which is consistent with findings that reduced circulating neutrophil counts are associated with T1D [25]. However, the neutrophil counts were comparable among the albuminuria patients with T1D and LADA, indicating that the degrees of inflammation involved in DKD in different types of diabetes may be similar. The logistic regression analysis showed neutrophil counts as an independent risk factor for DKD in T1D, but not in LADA, when performing multiple corrections. Expect for the possibility of insufficient sample size of LADA, another plausible explanation is that in T1D, hyperglycaemia usually starts in the first decades of life and is generally the only recognized cause of DKD in addition to inflammation. In contrast, in LADA, which is also an autoimmune diabetes but frequently accompanied by metabolic syndrome [12], hyperglycaemia usually develops after 30 years of age, when the kidneys begin suffering from the long-term consequences of ageing and other recognized promoters of chronic renal injury, such as older age, more obese, higher blood pressure, poor glycaemic control, bad lipid level control and severe albuminuria, which were presented by the LADA patients in our study, confounding the ability of inflammation in influencing DKD.

Recent epidemiologic studies showed a wide heterogenicity of DKD. In addition to classical albuminuric phenotype, there is a proportion of patients with decreased eGFR without albuminuria, the NA-DKD phenotype, in T1D and T2D [14, 26]. There were approximately one fifth (18.5%) of T1D patients with DKD, and approximately one third (37.9%) of LADA patients with DKD who had NA-DKD in our study. We found that neutrophil counts were an influencing factor for A-DKD in T1D, but not for NA-DKD in T1D. The pathogenesis of these phenotypes could be different [27], therefore future availability of better markers other than inflammatory indexes may shed more light in the pathogenesis of NA-DKD.

Our study has several limitations. First, the sample size of LADA patients was relatively small and the retrospective cross-sectional design cannot elucidate a causal link between leukocytosis and DKD. Future large-scale, longitudinal studies are needed to clarify the alterations and dynamic changes in the circulating neutrophil counts in the patients with different types of diabetes and to further investigate the concrete roles of the neutrophil counts in the pathogenesis of DKD in each type of diabetes. Second, this study did not provide serological data on neutrophil serine proteases, such as neutrophil elastase (NE), proteinase 3 (PR3), or cathepsin G (CG). Future studies focusing on whether neutrophils are activated to secrete the neutrophil serine proteases involved in the pathogenesis of DKD are warranted. Last, we did not assess changes in other inflammatory markers in this study; thus, it is necessary to further explore the underlying mechanisms of the contribution of inflammatory cytokines to DKD in autoimmune diabetes.

## Conclusions

Our study suggests that the neutrophil counts reflect DKD in subjects with autoimmune diabetes. These findings support the roles of neutrophils in the pathogenesis of the kidney complications of diabetes and provide a possible perspective for using neutrophils as a potential biomarker for the early identification of individuals at high risk of developing DKD and as potential therapeutic targets for DKD.

## Supplementary information

**Additional file 1.**

**Additional file 2.**

## Data Availability

The datasets used and analysed during the current study are available from the corresponding author on reasonable request.

## Abbreviations

ACR: Urinary albumin to creatinine ratio
A-DKD: Albuminuric DKD
BMI: Body mass index
CD11b: Cluster of differentiation molecule 11B
CG: Cathepsin G
CIs: Confidence intervals
CKD-EPI: Chronic Kidney Disease Epidemiology Collaboration formula
DBP: Diastolic blood pressure
DKD: Diabetic kidney disease
eGFR: Estimated glomerular filtration rate
ESRD: End-stage renal disease
FBG: Fasting glucose
FCP: Fasting C-peptide
HbA1c: Haemoglobin A1C
ICAM-1: Intercellular Adhesion Molecule 1
LADA: Latent autoimmune diabetes in adults
LDL-c: Low density lipoprotein cholesterol
MPO: Myeloperoxidase
NA-DKD: Non-albuminuric DKD
NE: Neutrophil elastase
NETs: Neutrophil extracellular traps
NLR: Neutrophil-to-lymphocyte ratio
ORs: Odds ratios
PBG: Postprandial glucose
PCP: 2 h Postprandial C-peptide
PR3: Proteinase 3
ROS: Reactive oxygen species
SBP: Systolic blood pressure
SPSS: Statistical Product and Service Solutions
T1D: Type 1 diabetes
TG: Triglyceride
WBCs: White blood cells
WC: Waist circles

## References

[1] Navarro-Gonzalez JF, Mora-Fernandez C, Muros de Fuentes M, Garcia-Perez J (2011). Inflammatory molecules and pathways in the pathogenesis of diabetic nephropathy. Nat Rev Nephrol.

[2] Wierusz-Wysocka B, Wykretowicz A, Byks H, Sadurska K, Wysocki H (1993). Polymorphonuclear neutrophils adherence, superoxide anion (O2-) production and HBA1 level in diabetic patients. Diabetes Res Clin Pract.

[3] Huang J, Xiao Y, Xu A, Zhou Z (2016). Neutrophils in type 1 diabetes. J Diabetes Invest.

[4] Azab B, Daoud J, Naeem FB, Nasr R, Ross J, Ghimire P, Siddiqui A, Azzi N, Rihana N, Abdallah M (2012). Neutrophil-to-lymphocyte ratio as a predictor of worsening renal function in diabetic patients (3-year follow-up study). Ren Fail.

[5] Huang W, Huang J, Liu Q, Lin F, He Z, Zeng Z, He L (2015). Neutrophil-lymphocyte ratio is a reliable predictive marker for early-stage diabetic nephropathy. Clin Endocrinol.

[6] Afsar B (2014). The relationship between neutrophil lymphocyte ratio with urinary protein albumin excretion in newly diagnosed patients with type 2 diabetes. Am J Med Sci.

[7] Ciray H, Aksoy AH, Ulu N, Cizmecioglu A, Gaipov A, Solak Y (2015). Nephropathy, but not Angiographically proven retinopathy, is associated with neutrophil to lymphocyte ratio in patients with type 2 diabetes. Exp Clin Endocrinol Diabetes.

[8] Akbas EM, Demirtas L, Ozcicek A, Timuroglu A, Bakirci EM, Hamur H, Ozcicek F, Turkmen K (2014). Association of epicardial adipose tissue, neutrophil-to-lymphocyte ratio and platelet-to-lymphocyte ratio with diabetic nephropathy. Int J Clin Exp Med.

[9] Kahraman C, Kahraman NK, Aras B, Cosgun S, Gulcan E (2016). The relationship between neutrophil-to-lymphocyte ratio and albuminuria in type 2 diabetic patients: a pilot study. Arch Med Sci.

[10] Wheelock KM, Saulnier PJ, Tanamas SK, Vijayakumar P, Weil EJ, Looker HC, Hanson RL, Lemley KV, Yee B, Knowler WC (2018). White blood cell fractions correlate with lesions of diabetic kidney disease and predict loss of kidney function in type 2 diabetes. Nephrol Dial ttransplant.

[11] Zhang J, Zhang R, Wang Y, Wu Y, Li H, Han Q, Guo R, Wang T, Wang J, Grung P (2019). Effects of neutrophil-lymphocyte ratio on renal function and histologic lesions in patients with diabetic nephropathy. Nephrology (Carlton).

[12] Zhou Z, Xiang Y, Ji L, Jia W, Ning G, Huang G, Yang L, Lin J, Liu Z, Hagopian WA (2013). Frequency, Immunogenetics, and clinical characteristics of latent autoimmune diabetes in China (LADA China study). Diabetes.

[13] Xiao Y, Liu L, Xu A, Zhou P, Long Z, Tu Y, Chen X, Tang W, Huang G, Zhou Z (2015). Serum fibroblast growth factor 21 levels are related to subclinical atherosclerosis in patients with type 2 diabetes. Cardiovasc Diabetol.

[14] Laranjinha I, Matias P, Mateus S, Aguiar F, Pereira P, Perneta Santos M, Costa R, Lourenco A, Guia J, Barata JD (2016). Diabetic kidney disease: is there a non-albuminuric phenotype in type 2 diabetic patients?. Nefrologia.

[15] Deckert T, Poulsen J (1981). Diabetic nephropathy: fault or destiny?. Diabetologia.

[16] Takayuki F, Seiichiro H, Mamiko K, Minako Y, Yoshinobu F, Atsushi S, Masayoshi S (2013). Complement-mediated chronic inflammation is associated with diabetic microvascular complication. Diabetes Metab Res Rev.

[17] Fardon NJ, Wilkinson R, Thomas TH (2002). Abnormalities in primary granule exocytosis in neutrophils from type I diabetic patients with nephropathy. Clin Sci (Lond).

[18] Takahashi T, Hato F, Yamane T, Inaba M, Okuno Y, Nishizawa Y, Kitagawa S (2000). Increased spontaneous adherence of neutrophils from type 2 diabetic patients with overt proteinuria: possible role of the progression of diabetic nephropathy. Diabetes Care.

[19] Michelis R, Kristal B, Zeitun T, Shapiro G, Fridman Y, Geron R, Sela S (2013). Albumin oxidation leads to neutrophil activation in vitro and inaccurate measurement of serum albumin in patients with diabetic nephropathy. Free Radic Biol Med.

[20] Elena G, Klaus L (2006). Leukocyte recruitment and vascular injury in diabetic nephropathy. J Am Soc Nephrol.

[21] Wong SL, Demers M, Martinod K (2015). Diabetes primes neutrophils to undergo NETosis, which impairs wound healing. Nat Med.

[22] Yudong W, Yang X, Ling Z, Dewei Y, Jialiang Z, Yiting T, Bornstein SR, Zhiguang Z, Lam KSL, Aimin X (2014). Increased neutrophil elastase and proteinase 3 and augmented NETosis are closely associated with β-cell autoimmunity in patients with type 1 diabetes. Diabetes.

[23] Ojha S, Alkaabi J, Amir N (2014). Withania coagulans fruit extract reduces oxidative stress and inflammation in kidneys of streptozotocin-induced diabetic rats. Oxid Med Cell Longev.

[24] Kawamoto R, Ninomiya D, Kikuchi A, Akase T, Kasai Y, Kusunoki T, Ohtsuka N, Kumagi T (2019). Association of neutrophil-to-lymphocyte ratio with early renal dysfunction and albuminuria among diabetic patients. Int Urol Nephrol.

[25] Huang J, Xiao Y, Zheng P, Zhou W, Wang Y, Huang G, Xu A, Zhou Z (2019). Distinct neutrophil counts and functions in newly diagnosed type 1 diabetes, latent autoimmune diabetes in adults, and type 2 diabetes. Diabetes Metab Res Rev.

[26] Molitch ME, Steffes M, Sun W, Rutledge B, Cleary P, de Boer IH, Zinman B, Lachin J, Epidemiology of diabetes I, complications study G (2010). Development and progression of renal insufficiency with and without albuminuria in adults with type 1 diabetes in the diabetes control and complications trial and the epidemiology of diabetes interventions and complications study. Diabetes Care.

[27] Chawla V, Roshan B (2014). Non-proteinuric diabetic nephropathy. Curr Diab Rep.

