# Supplementary material for: Neutrophil count as a reliable marker for diabetic kidney disease in autoimmune diabetes
Source: BMC Endocr Disord. 2020 Oct 22;20:158. doi: 10.1186/s12902-020-00597-2 (PMC7580021; doi:10.1186/s12902-020-00597-2)
Supplement: Supplementary file 2 — Additional file 2. [file 12902_2020_597_MOESM2_ESM.docx]

Supplementary Table 1. Binary logistic regression analysis showing risk factors for albuminuric diabetic kidney disease.

|  |  | T1D | | | | |  |  | LADA | | | | | |  | |  |
| --- | --- | --- | --- | --- | --- | --- | --- | --- | --- | --- | --- | --- | --- | --- | --- | --- | --- |
|  | Simple | Multiple | |  | | |  | Simple | Multiple | |  | | | | | |  |
|  | P | OR | P | | 95%CI | |  | P | | OR | | P | 95%CI | | |  |  |
|  |  |  |  | | LCI | UCI |  |  | |  | |  | LCI | UCI | | |  |
| Age | 0.008** | 1.036 | 0.074 | | 0.997 | 1.076 |  | 0.062 | | 1.004 | | 0.936 | 0.921 | 1.093 | | |  |
| Gender | 0.263 | 3.316 | 0.073 | | 0.896 | 12.271 |  | 0.778 | | 1.562 | | 0.706 | 0.154 | 15.837 | | |  |
| Duration | 0.001** | 1.060 | 0.227 | | 0.964 | 1.165 |  | 0.002** | | 1.179 | | 0.022* | 1.024 | 1.356 | | |  |
| BMI | 0.132 | 1.009 | 0.934 | | 0.811 | 1.255 |  | 0.260 | | .802 | | 0.187 | 0.577 | 1.113 | | |  |
| HbA1c | 0.090* | .599 | 0.040* | | 0.367 | 0.978 |  | 0.604 | | 1.393 | | 0.169 | 0.868 | 2.236 | | |  |
| FBG | 0.877 | 1.052 | 0.492 | | 0.910 | 1.218 |  | 0.441 | | .853 | | 0.318 | 0.625 | 1.165 | | |  |
| FCP | 0.449 | 1.002 | 0.697 | | 0.994 | 1.009 |  | 0.828 | | .999 | | 0.869 | 0.992 | 1.007 | | |  |
| Hypertension | 0.647 | .684 | 0.516 | | 0.218 | 2.150 |  | 0.003** | | 15.720 | | 0.011* | 1.888 | 130.883 | | |  |
| Dyslipidemia | 0.202 | .481 | 0.295 | | 0.122 | 1.893 |  | 0.741 | | 1.925 | | 0.487 | 0.304 | 12.212 | | |  |
| N | 0.004** | 1.844 | 0.021* | | 1.098 | 3.098 |  | 0.017* | | 1.100 | | 0.867 | 0.360 | 3.359 | | |  |
| Smoking | 0.602 | 2.801 | 0.088 | | 0.859 | 9.132 |  | 0.109 | | 10.618 | | 0.058 | 0.923 | 122.187 | | |  |

Abbreviations: T1D, type 1 diabetes; LADA, latent autoimmune diabetes in adults; N, neutrophil counts; BMI, body mass index; FBG, fasting blood glucose; FCP, fasting C-peptide; *p<0.05; **p<0.01.

Supplementary Table 2. Binary logistic regression analysis showing risk factors for non-albuminuric diabetic kidney disease.

|  | T1D | | | | |  |  | | LADA | | | | | |  | | | |
| --- | --- | --- | --- | --- | --- | --- | --- | --- | --- | --- | --- | --- | --- | --- | --- | --- | --- | --- |
|  | Simple | Multiple | | | |  |  | | Simple | | Multiple | | | | | | |  |
|  | P | OR | P | 95%CI | |  | | P | | OR | | P | 95%CI | | |  |  |  |
|  |  |  |  | LCI | UCI |  | |  | |  | |  | LCI | UCI | | |  |  |
| Age | 0.003** | 1.261 | 0.057 | 0.993 | 1.601 |  | | 0.002** | | 1.450 | | 0.016* | 1.071 | 1.963 | | |  |  |
| Gender | 0.726 | 39.695 | 0.152 | 0.259 | 6077.742 |  | | 0.123 | | 33.477 | | 0.036* | 1.251 | 895.829 | | |  |  |
| Duration | 0.057 | 1.007 | 0.957 | 0.773 | 1.313 |  | | 0.270 | | 0.818 | | 0.083 | 0.652 | 1.027 | | |  |  |
| BMI | 0.453 | 0.646 | 0.299 | 0.283 | 1.475 |  | | 0.929 | | 0.589 | | 0.081 | 0.325 | 1.067 | | |  |  |
| HbA1c | 0.695 | 0.224 | 0.176 | 0.026 | 1.956 |  | | 0.324 | | 0.368 | | 0.058 | 0.131 | 1.034 | | |  |  |
| FBG | 0.275 | 0.888 | 0.637 | 0.542 | 1.455 |  | | 0.787 | | 0.942 | | 0.704 | 0.690 | 1.285 | | |  |  |
| FCP | 0.423 | 0.983 | 0.454 | 0.939 | 1.028 |  | | 0.537 | | 1.002 | | 0.747 | 0.992 | 1.011 | | |  |  |
| Hypertension | 0.456 | 0.199 | 0.577 | 0.001 | 57.346 |  | | 0.941 | | 0.018 | | 0.048* | 0.000 | 0.968 | | |  |  |
| Dyslipidemia | 0.293 | 101.394 | 0.237 | 0.048 | 213090.581 |  | | 0.447 | | 21.040 | | 0.087 | 0.642 | 689.372 | | |  |  |
| N | 0.365 | 0.557 | 0.530 | 0.090 | 3.462 |  | | 0.304 | | 0.328 | | 0.170 | 0.067 | 1.614 | | |  |  |

Abbreviations: T1D, type 1 diabetes; LADA, latent autoimmune diabetes in adults; N, neutrophil counts; BMI, body mass index; FBG, fasting blood glucose; FCP, fasting C-peptide; *p<0.05; **p<0.01.
